# Interactions between Cognition and Hearing Aid Compression Release Time: Effects of Linguistic Context of Speech Test Materials on Speech-in-Noise Performance

**DOI:** 10.3390/audiolres11020013

**Published:** 2021-04-02

**Authors:** Jingjing Xu, Robyn M. Cox

**Affiliations:** School of Communication Sciences and Disorders, University of Memphis, Memphis, TN 38152, USA; robyncox@memphis.edu

**Keywords:** speech recognition, cognition, linguistic context, release time, hearing aids

## Abstract

Recent research has established a connection between hearing aid (HA) users’ cognition and speech recognition performance with short and long compression release times (RT). Contradictive findings prevent researchers from using cognition to predict RT prescription. We hypothesized that the linguistic context of speech recognition test materials was one of the factors that accounted for the inconsistency. The present study was designed to examine the relationship between HA users’ cognition and their aided speech recognition performance with short and long RTs using materials with various linguistic contexts. Thirty-four older HA users’ cognitive abilities were quantified using a reading span test. They were fitted with behind-the-ear style HAs with adjustable RT settings. Three speech recognition tests were used: the word-in-noise (WIN) test, the American four alternative auditory feature (AFAAF) test, and the Bamford-Kowal-Bench speech-in-noise (BKB-SIN) test. The results showed that HA users with high cognitive abilities performed better on the AFAAF and the BKB-SIN than those with low cognitive abilities when using short RT. None of the speech recognition tests produced significantly different performance between the two RTs for either cognitive group. These findings did not support our hypothesis. The results suggest that cognition might not be important in prescribing RT.

## 1. Introduction

Digital, wide dynamic range compression (WDRC) hearing aids, which provide nonlinear amplification, have been increasingly used nowadays. Release time (RT) is an important parameter of the nonlinear compression function, determining how quickly the compressor reacts to a decrease in input sound level. RT varies from milliseconds to seconds. There is no established definition regarding short and long RTs. It is generally accepted that RTs can be considered short when they are less than 100 milliseconds and long when they are greater than 500 milliseconds. With different RT settings, temporal envelopes of amplified sounds, especially speech signals, can vary drastically. The advantages and disadvantages for short and long RTs were thoroughly reviewed and briefly summarized here [1].

A compressor with a short RT reacts very quickly to changes in sound intensity. It has been claimed that it allows the hearing aid to provide greater gain to soft sounds, which results in an improvement in audibility of soft consonants and thereby, to some extent, restores loudness perception to “normal.” Despite the merits of short RT processing, some potential drawbacks are also evident. By providing more gain to softer sounds, short-term amplitude contrasts of a speech signal (e.g., consonant-to-vowel ratio) will be altered, and the temporal pattern of the speech signal will be distorted. As a consequence, the naturalness of the processed speech signal will be compromised. Another frequently claimed disadvantage in using short RT processing is the perceived distraction with background noise during ongoing speech pauses, which deteriorates listening comfort.

A compressor with long RT processing maintains gain for a longer period of time compared to a compressor with short RT processing. Thus, the internal intensity difference of a speech signal can be largely preserved, and minimum perceived distortion can be achieved. As a result, speech signals processed with a long RT could be more natural, and the listening comfort of hearing aid wearers could be increased. Similarly, with a long RT, the larger gain will not be applied to the relatively low-level background noise during the pauses of speech, and consequently, listening comfort could be improved. Adverse effects of long RT processing are also substantial. First, available speech cues in a modulated background masker may not receive enough amplification with long RT processing, so that speech understanding ability in such a condition will be diminished. Second, when an intense sound precedes a softer sound, the gain applied to the intense sound will be decreased due to the nonlinear amplification algorithm. Such a lower amount of gain will still be applied to the succeeding softer sounds because of a long compression release period, which may result in inaudibility.

Unfortunately, to date, no standardized strategy or protocol for prescribing RT exists. Audiologists and hearing aid specialists often use the default RT setting that is recommended by the hearing aid’s manufacturer. With this method, not all hearing aid users are satisfied.

The importance of RT selection has been well acknowledged, and several studies have sought to determine the overall superiority of either short or long RT (e.g., [2,3]). Such previous studies have investigated the advantages and disadvantages of different RTs under a variety of test conditions. Findings have been inconclusive, and numerous factors could possibly account for the discrepant results.

More recent research has examined the role of listeners’ cognition in determining RT superiority [4,5,6,7,8,9,10,11]. Researchers observed that cognitive functions that were closely associated with speech recognition performance were related to working memory capacity, which includes temporal storage and central executive [12,13]. The temporal storage function of working memory allows auditory information to be accumulated, while the central executive system of working memory decodes and processes the accumulated information. Deterioration of either of these two functions will very likely compromise the efficiency and accuracy of communication and intellectual abilities, especially for older listeners [14]. Because working memory capacity and RT are both related to speech recognition performance, an investigation of the interaction between these two variables has received great research attention.

A seminal paper by Gatehouse et al. [7] explored the relationship between cognition and aided speech recognition performance with short and long RTs. In Gatehouse’s study, hearing aid users’ cognitive abilities were evaluated using a visual letter monitoring test [6]. Their aided speech recognition performance when using short and long RT settings (40 ms versus 640 ms) was evaluated using the four alternative auditory feature (FAAF) test [15] after a 10 week acclimatization period for each RT setting [6]. The correlation between the benefit of short over long RT and cognitive scores was examined, and the correlation coefficient was 0.30. Based on this correlation, Gatehouse and colleagues suggested that listeners with greater cognitive abilities perform better with short RTs, while listeners with poorer cognitive abilities perform better with long RTs. This finding has shed important light on the role of cognition in aided hearing and inspired researchers to further investigate this topic.

Later studies on this topic used the same hearing aid technologies and compared the same RT settings. Among these studies, Gatehouse’s finding was bolstered by Lunner and Sundewall-Thoren [8] and Rudner et al. [11], which included the same cognitive test, but used different speech recognition tests in different languages. However, Gatehouse’s finding could not be replicated by other studies [4,5] with a similar research design. They found that RT is crucial only for hearing aid users with low cognitive abilities.

Why cannot Gatehouse’s finding be replicated in some studies? Cox and Xu [4] suggested that one factor that could potentially underlie the inconsistent findings is the linguistic context of speech test materials. In these studies, different speech recognition test materials have been used. Some studies have used sentence tests (e.g., [5]), whereas some have used word-based tests (e.g., [7]). Different test materials provide different amounts of linguistic context. Target words in test materials with high linguistic context are highly predictable, whereas target words in test materials with low linguistic context are less likely or even impossible to be predicted. Linguistic contexts simply provide an additional source of information that supplements the sensory information [16]. Thus, when using speech recognition test materials with high linguistic context, top-down processing may be engaged to assist in speech understanding in addition to bottom-up processing resulting in audibility. There is evidence that the ability to use linguistic context in assisting speech understanding is associated, at least to some extent, with hearing aid users’ working memory capacity [17]. Therefore, it is reasonable to expect that the linguistic context of speech recognition test materials impacts the relationship between hearing aid wearers’ cognitive abilities (primarily working memory capacity) and their aided speech recognition performance with different RTs.

The primary goal of the current study was to further extend the line of investigation to address the relationship between cognitive abilities of listeners with hearing impairment and aided speech understanding performance with varying RTs when speech recognition tests with different amount linguistic contexts were used. The specific research questions were as the following:

(1) Is the finding reported in Gatehouse et al.’s [7] study replicable using an equivalent speech recognition test?
(2) What is the relationship between cognitive abilities and aided speech recognition performance in noise with short and long release times?
(3) What is the effect of the linguistic context of speech recognition test material on measuring this relationship?

## 2. Materials and Methods

The present study was a double-blinded nonrandomized intervention study, and it was approved by the Institutional Review Board of the University of Memphis.

### 2.1. Participants

**Figure 1:**
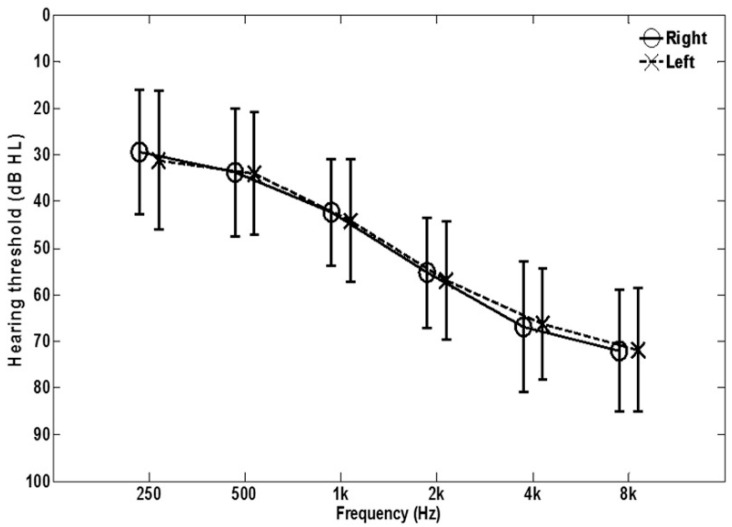
Mean audiogram for the 34 participants. Error bars show ± 1 SD.

Thirty-four experienced hearing aid users (20 males and 14 females) participated in the present study. They were all bilateral hearing aid users except two. Their age range was from 54 to 91 (M = 73.6, SD = 9.3). The participants had essentially symmetrical mild to moderate sensorineural hearing loss (pure-tone average differences between two ears ≤ 15 dB). Mean hearing thresholds are shown in Figure 1. These participants were recruited from the University of Memphis Hearing Aid Research Laboratory subject database and the Memphis Speech and Hearing Center clinic. The participants who completed the study were monetarily compensated for their participation.

### 2.2. Hearing Aids

**Figure 2:**
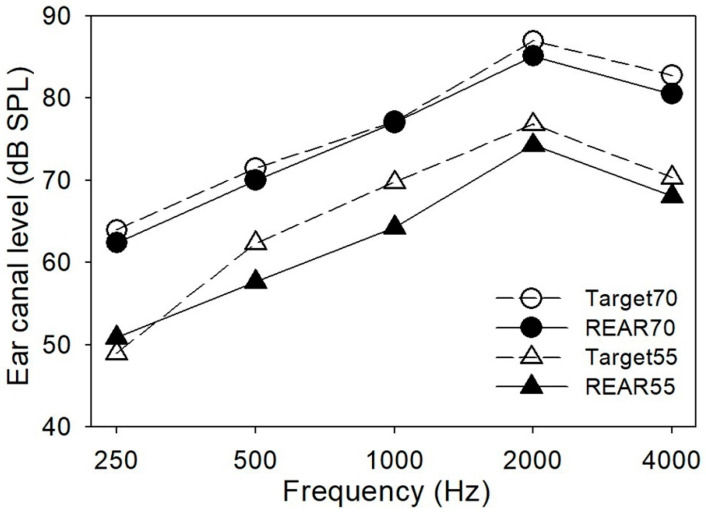
Mean real-ear-aided response (REAR) values for 55 dB SPL and 70 dB SPL speech compared to the NAL-NL1 targets across the 34 participants (68 ears).

Starkey S Series 9 behind-the-ear style hearing aids were bilaterally fitted to each participant with closed earmolds using the National Acoustic Laboratories’ nonlinear fitting procedure, version 1 (NAL-NL1) prescription method [18]. These hearing aids were 12-channel digital WDRC devices with adjustable compression time constants. The nominal compression time constant settings (attack/release) for short and long processing were: 15/50 milliseconds and 20/2000 milliseconds, respectively. This hearing aid type also provided some advanced features, such as directional microphones, digital noise reduction, and feedback cancellation. However, all advanced features except feedback cancellation were inactivated. In addition, as many as four memories were available for different environments and directional settings. Only one memory was used. Mean hearing aid fitting data are shown in Figure 2.

It is important to note that the nominal time constants are determined by an engineering method using electrical circuit parameters. It was worth measuring the actual time constants for each fitting to ensure that these two-time constant settings did provide a considerable RT difference. For this purpose, time constants were measured using a Fonix 7000 hearing aid test system (Frye Electronics Inc., Beaverton, OR, USA) after each fitting. Mean RT was calculated for hearing aids across frequencies from 500 to 5000 Hz. The mean short and long RTs were 126 ms and 938 ms, respectively. Even though the short and long RTs measured in the test box were not equivalent to the nominal values specified by the manufacturer, there was still a substantial difference between the two RT settings.

### 2.3. Cognitive Test

A reading span test [19,20] was used to quantify hearing aid users’ cognitive abilities. This test comprised 54 test sentences and three practice sentences. The test sentences were presented in groups that range in size from three to six sentences per group. There were three groups of sentences for each of the four sizes. Each sentence was composed of three categories: a person, a verb, and an object. Among all test sentences, half of the sentences were nonsense sentences, and the other half were normal sentences. The software displayed the sentences on a computer monitor in a word-by-word fashion. The participants were instructed to respond “yes” to the sentences, which were normal and respond “no” to sentences, which were nonsense after each sentence. After each group of sentences, the tester said either “first” or “last,” indicating that the participant should start to recall either the first or the final words of each previously presented sentence in their correct serial order. The order of recalling the first or final words was randomized. The three practice sentences served as a practice group before actual tests. In the testing phase, the test sentences were presented in groups in ascending order until the last group with six sentences was completed. The percentage of the words that were correctly recalled was the performance measure.

### 2.4. Speech Recognition Tests

Three speech recognition tests, which differ in the amount of linguistic context, were used. They were the words-in-noise (WIN) test [21], the Bamford-Kowal-Bench speech-in-noise (BKB-SIN) test (Etymotic Research, 2005), and the American four alternative auditory feature (AFAAF) test [15,22].

#### 2.4.1. WIN Test

The WIN test that was used in the current study had been modified based on the WIN material from the Speech recognition and identification materials, Disc 4.0. CD (Department of Veterans Affairs, 2006). There are four randomizations, each of which has 70 target words from the NU6 test [23]. Each target word is presented with a carrier phrase: “Say the word___.” The masking noise is a six-talker babble. The speech level decreases with a constant masker level to form seven SNR conditions from +24 dB to 0 dB in 4 dB steps. In this study, the noise level was fixed at 50 dB SPL. There were 20 target words for the score at each SNR. The WIN is an open-set word-based test, and the effects of working memory and inter-word context on recognition performance are minimized [24]. This test provides minimal linguistic context, so no prediction is possible. Therefore, the WIN test is considered a context-low material.

#### 2.4.2. BKB-SIN Test

The BKB-SIN test is a sentence test, which is composed of context-rich sentences presented with a four-talker babble masker. In this study, only list pairs one to eight in the BKB-SIN CD one were used. The target talker and background babble of each of the eight pairs are recorded on the same channel at 10 pre-recorded SNRs from +21 dB to -6 dB with a step size of 3 dB for each sentence. In this study, the speech level was calibrated at 65 dB SPL for +21 dB SNR. There were 24 target words per SNR. The BKB-SIN test is an open-set sentence test, and the contextual cues within each test sentence are substantial. Therefore, the BKB-SIN test is considered a context-rich material.

#### 2.4.3. AFAAF Test

The AFAAF test is a word-based closed-set test. It is an American dialect version of the FAAF test [15]. It comprises 80 test words and five practice words. In each utterance, a monosyllabic keyword is embedded in a carrier sentence: “Can you hear ____ clearly?” For example, the test utterance is: “Can you hear OLD clearly?” and the four alternatives displayed on a computer monitor are “HOLD”, “OLD”, “COLD”, and “GOLD”. The performance measure is the percentage of the keywords that are correctly identified.

For this study, the 80 test words were divided into four lists of 20 words each. The four lists were equivalent concerning consonant place (initial versus final) and difficulties in consonant recognition (based on unpublished data). The five practice words were provided at the very beginning of the test to familiarize the participant with the task. In this study, an amplitude-modulated noise was used as the masker. This noise (ICRA CD, track 7; [25]) was a talker-matched speech spectrum noise modulated by a six-talker babble envelope. Participants were tested under eight SNR conditions from −9 to 12 dB SNR in 3 dB steps. Speech level was fixed to 65 dB SPL for SNR conditions from 0 to +12 dB SNR. The masking noise level was fixed to 65 dB SPL for SNR conditions from −9 to −3 dB SNR. One 20-word list was used for each SNR condition. Because the AFAAF keywords use the same carrier sentence, it is considered low in linguistic context. However, due to its closed-set nature, some predictive clues (e.g., phonological cues) may be available from the displayed alternatives.

### 2.5. Procedure

All tests were administered in the University of Memphis Hearing Aid Research Laboratory. Two sessions were required for each participant.

In session one, a hearing evaluation was carried out for obtaining information about the participant’s current hearing loss and unaided speech recognition abilities. Then, a reading span test was given to assess the participant’s cognitive abilities. After this, hearing aids were fitted bilaterally to the participant. There was no real-world acclimatization period prior to testing to maximize the effects of different RTs [26].

In session two, the participant’s aided speech recognition performance with the first RT setting was evaluated under the SNR conditions using three speech recognition tests. After completing all speech tests with the first RT setting, the second RT setting was programmed to both hearing aids. The same speech recognition tests were administered again with different test lists. Counterbalancing controlled the order of presenting the three speech recognition tests. Test lists of each speech recognition test were also counterbalanced or randomized to minimize order effects. In addition to an immediate scoring, each participant’s responses were recorded with a digital recorder. A research assistant who had no idea of the research design scored the two open-set speech recognition tests after each appointment for intra-judge scoring reliability.

All speech recognition testing took place in a double-walled sound room. Audio signals from the computer soundcard were routed through a GSI-61 audiometer. Speech and noise signals from the audiometer were mixed into one channel and amplified by an ASHLY PE-800 external amplifier, and then delivered to a Boston Acoustics CR57 loudspeaker. The participants were seated in the sound room, one meter from the loudspeaker.

### 2.6. Statistical Analysis

With each speech recognition test, a participant had one score in percent correct for each SNR with each RT. For each participant, a psychometric function was fitted to the discrete percentage correct scores for each RT on each test. For the BKB-SIN test and the WIN test, the potential fitting range was from 0% to 100%. The psychometric functions were best-fit, three-parameter sigmoid functions (see Equation (1)):

(1) $$y=\frac{a}{1+e^{-\frac{x-b}{c}}}$$

where *x* was SNR; *y* was the speech recognition performance in percent correct as a function of SNR; *a*, *b*, and *c* were parameters of the sigmoid function. The values of these three parameters were changed when this equation was used to curve-fit different sets of discrete data. For the AFAAF test, however, the potential fitting range was from 25% to 100% due to the chance performance of its four-alternative closed-set test format. Thus, Equation (1) was modified for the AFAAF data (see Equation (2)):

(2) $$y=\frac{a}{1+e^{-\frac{x-b}{c}}}+25$$

The variable for statistical analysis was the SNR value at 50% correct point (SNR50) on the participant’s psychometric function. This was the performance in a quite challenging situation. The rationale for selecting this performance measure was to ensure the engagement of working memory when the listening task was sufficiently taxing for the listener [27].

For assessing the relationship between cognitive abilities, RT, and linguistic context, mixed model repeated measures ANOVAs were used. SNR50 was the dependent variable. The full model ANOVA included two within-subject variables: RT (short vs. long) and context (the three speech recognition tests), and one between-subject variable, group (low and high cognitive performance). In addition, other mixed model repeated measure ANOVAs were performed on subsets of the data to further analyze the interaction between RT and group for each speech recognition test. These analyses were performed using Statistical Package for the Social Sciences (SPSS) Version 16 software. For all analyses, any *p*-value of 0.05 or lower is considered statistically significant, while *p*-values between 0.05 and 0.1 are considered marginally significant. With 34 participants, this study had > 80% power to detect a medium effect (effect size f = 0.25, see [28]) of RT on speech recognition scores for the interaction between RT and cognitive ability at a significance level of 0.05 using G*power 3 programs [29].

## 3. Results

### 3.1. Cognitive Data and Speech Recognition Data

**Figure 3:**
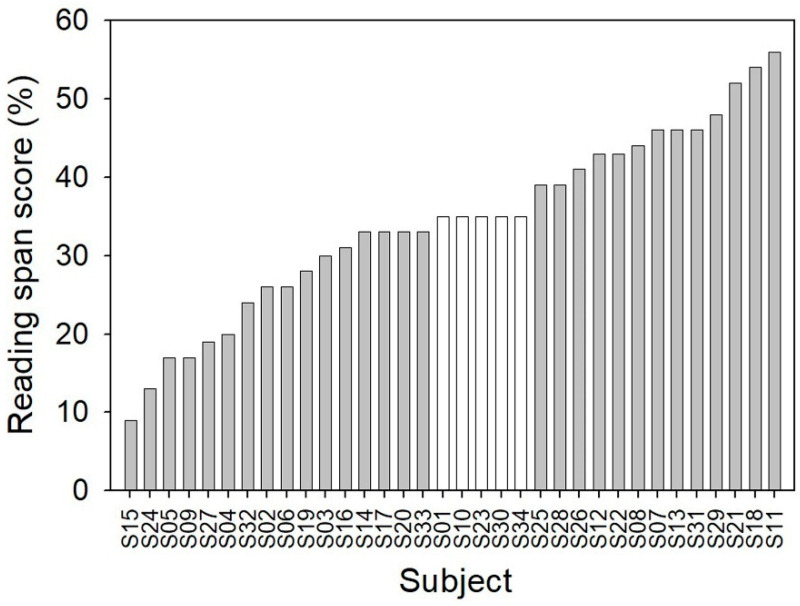
Reading span scores for all 34 participants (S01 to S34). For other analyses presented later, two cognitive performance groups were created by excluding the five participants with their reading span scores of 35 (white bars). Among the remaining 29 participants (gray bars), those whose reading span scores were lower than 35 were considered cognitively low-performance participants (16 participants), and those whose reading span scores were higher than 35 were considered cognitively high-performance participants (13 participants).

**Figure 4:**
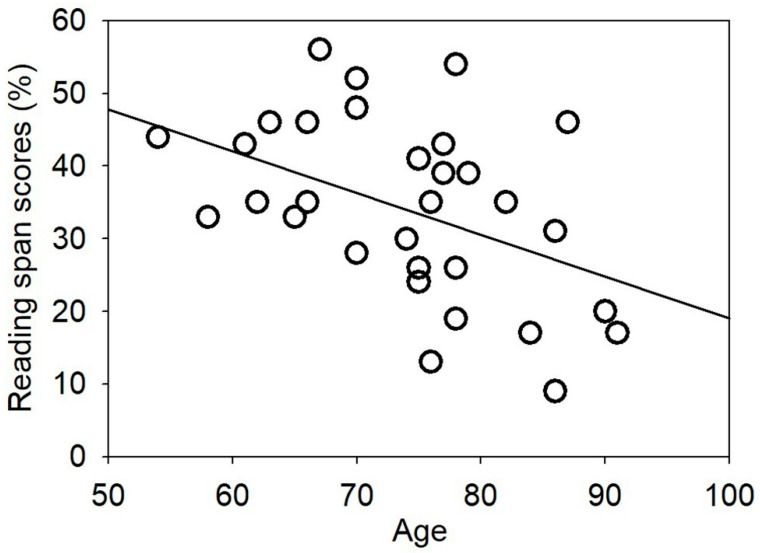
Scatter plot showing the correlation between the participants’ reading span scores and their age (r = −0.453). The straight line is the regression line.

The obtained reading span scores ranged from 9 to 56. The average score across the 34 participants was 34.2 (SD = 11.8). Scores for all of the participants were ranked from low to high and plotted in Figure 3. A Pearson’s product-moment correlation analysis showed that the participants’ reading span scores were inversely correlated to their ages (r = −0.453, *p* = 0.007). A scatter plot depicts this relationship (Figure 4).

Regarding speech recognition performance, mean SNR50 values and the corresponding standard deviations for the three speech recognition tests are listed in Table 1. Note that a lower SNR50 means better speech recognition performance.

### 3.2. Correlation between Cognitive Abilities and Benefit of Short RT

In the present study, we replicated the procedures used in [7] to examine the correlation between reading span score and benefit of short over long RT (Benefit-ShortRT) with different speech recognition tests and hearing aids with more compression channels. The Benefit-ShortRT score was computed for each participant by subtracting the SNR50 value for the short RT from the SNR50 value for the long RT in each speech recognition test. Thus, a positive value of Benefit-ShortRT indicated that performance was better with the short RT, while a negative value indicated performance was better with the long RT. A data cleaning procedure on the Benefit-ShortRT scores revealed that the highest benefit values for the AFAAF test and the BKB-SIN test were true outliers, which violated the assumptions of Pearson’s product–moment correlation. One-way of minimizing the impact of outliers is to change the value(s) of the variable(s) for the outlying case(s) so that they are deviant but not as deviant as they were [30]. In the present study, the extreme value in each of the two speech recognition tests was changed to a value that was one unit greater than the next most extreme value in the distribution.

**Figure 5:**
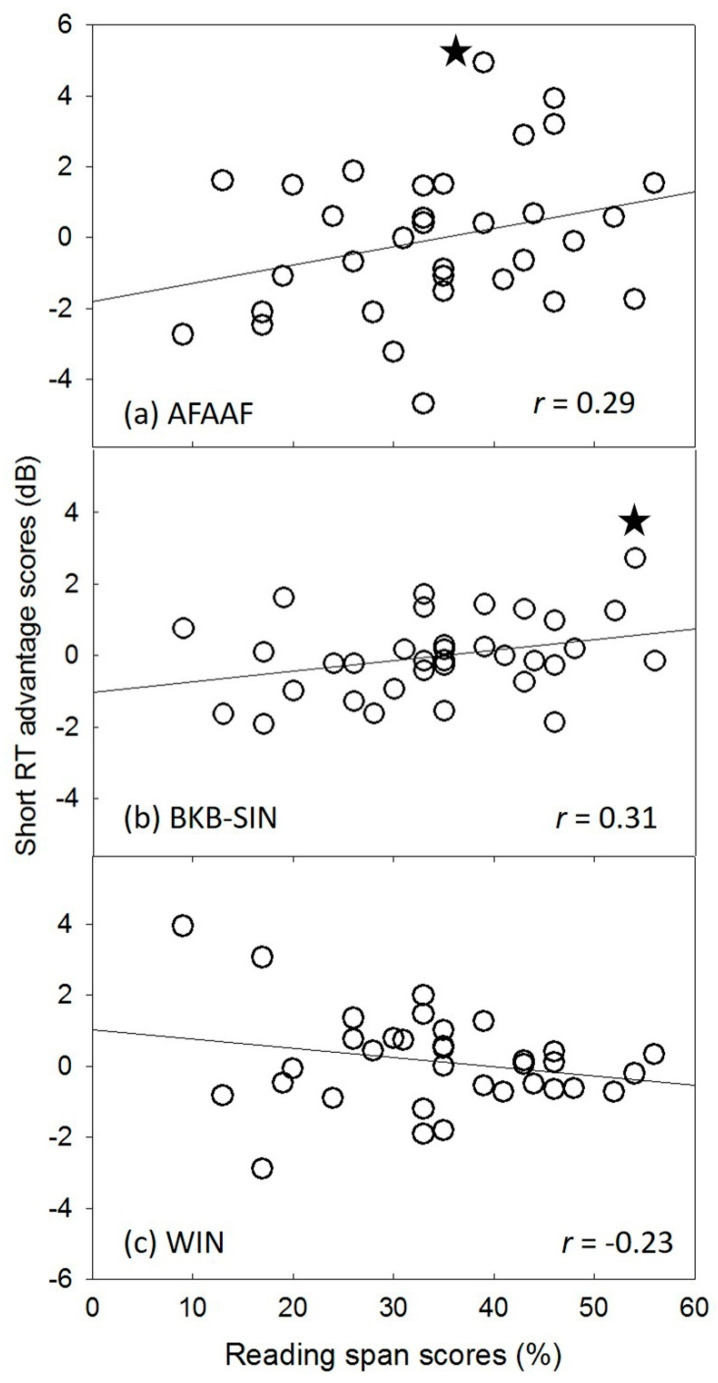
Scatter plots showing the relationship between the reading span scores and Benefit-ShortRT scores for the three speech recognition tests: (**a**) American four alternative auditory feature (AFAAF) test; (**b**) Bamford-Kowal-Bench speech-in-noise (BKB-SIN) test; (**c**) word-in-noise (WIN) test. The solid line in each of the sub-plots is the regression line. The star symbols indicate the data points that were adjusted.

Figure 5 shows the scatter plots depicting the correlation between Benefit-ShortRT scores and the reading span scores for each speech recognition test. The Pearson’s product–moment correlation coefficients for the AFAAF test and the BKB-SIN test (r_AFAAF_ = 0.29, *p* = 0.097; r_BKB-SIN_ = 0.31, *p* = 0.076) were similar to the one reported in the study conducted by Gatehouse (r = 0.30). By contrast, the Benefit-ShortRT scores obtained from the WIN were negatively correlated with the reading span scores (r_WIN_ = −0.23, *p* = 0.194), which was inconsistent with Gatehouse’s finding.

### 3.3. Creating Two Cognitive Performance Groups

**Figure 6:**
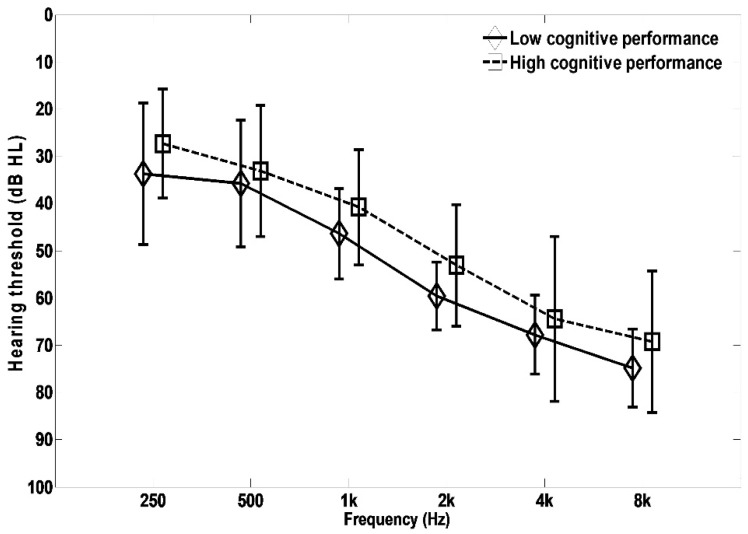
Mean audiograms for the low and the high cognitive performance groups. In each group, hearing thresholds for the left and the right ears were combined.

To assess the difference between the two RTs for participants with low and high cognitive abilities, two cognitive groups were created by excluding the five participants from the middle of the reading span score distribution (Figure 3). The age range for the participants in the cognitively low-performance group was from 58 to 91 years (M = 76.0, SD = 9.7), while the age range for the participants in the cognitively high-performance group was from 54 to 87 years (M = 71.1, SD = 8.9). The age difference between the two groups was not statistically significant (t (27) = 1.406, *p* = 0.171). The mean audiograms for the two groups are depicted in Figure 6. It is seen that the participants in the cognitively low-performance group have 5 to 10 dB more hearing impairment on average than the participants in the cognitively high-performance group across frequencies (F (1,27) = 3.595, *p* = 0.069). Psychometric functions were fitted to the mean percent correct scores for each cognitive group and each speech recognition test with both RTs using Equations (1) and (2) for displaying performance patterns (Appendix A).

### 3.4. Speech Recognition Data with Different Tests and RTs in Each Cognitive Group

**Figure 7:**
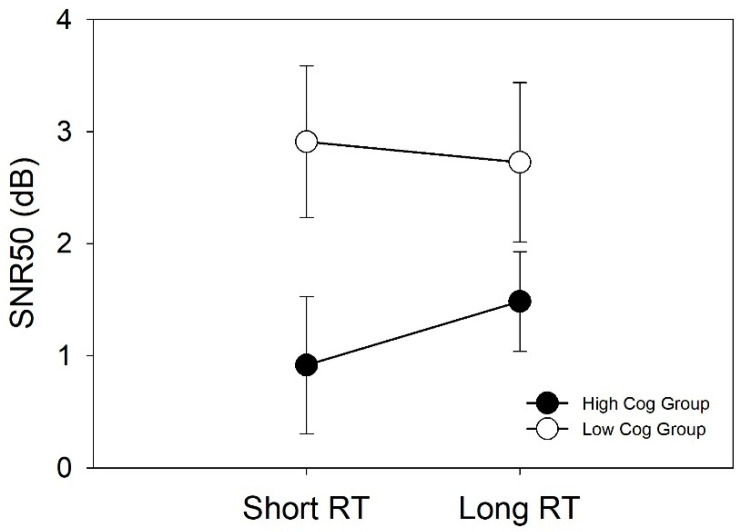
Interaction between cognitive performance group and RT when mean SNR50 scores of the three speech recognition tests were combined. Error bars represent standard errors.

Table 2 shows the main effects of the three variables (RT, context, and group) and their interactions based on the full model ANOVA. The two-way interaction between cognitive abilities and RT was marginally significant (F(1,27) = 4.023, *p* = 0.055). Figure 7 depicts this interaction. The overall pattern shows that participants in the high cognitive group had better speech recognition performance than those in the low cognitive group. Post hoc pair-wise comparisons revealed (1) a marginally significant effect that the participants with high cognitive abilities performed better with a short RT than with a long RT (*p* = 0.051), and (2) a significant effect that the participants in the high cognitive group performed better than those in the low cognitive group when using the short RT (*p* = 0.042). However, the mean SNR50 difference between the two RTs for either group is less than 1 dB.

The three-way interaction (F (1.609,43.448) = 3.303, *p* = 0.056) was further analyzed. A mixed model ANOVA was performed for each speech recognition test to examine the interaction effect between group and RT. Table 3 shows the mean SNR50 scores for the two RTs and the two cognitive groups. Analysis results showed that the main effect of RT was not statistically significant for the AFAAF test (F (1,27) = 0.483, *p* = 0.493), the BKB-SIN test (F (1,27) = 0.215, *p* = 0.647), or the WIN test (F (1,27) = 0.247, *p* = 0.623). Furthermore, the results showed a marginally significant main effect of cognitive group for the AFAAF test (F (1,27) = 3.876, *p* = 0.059) and the BKB-SIN test (F (1,27) = 4.167, *p* = 0.051). However, the main effect of cognitive group was not statistically significant for the WIN test (F (1,27) = 1.128, *p* = 0.298).

**Figure 8:**
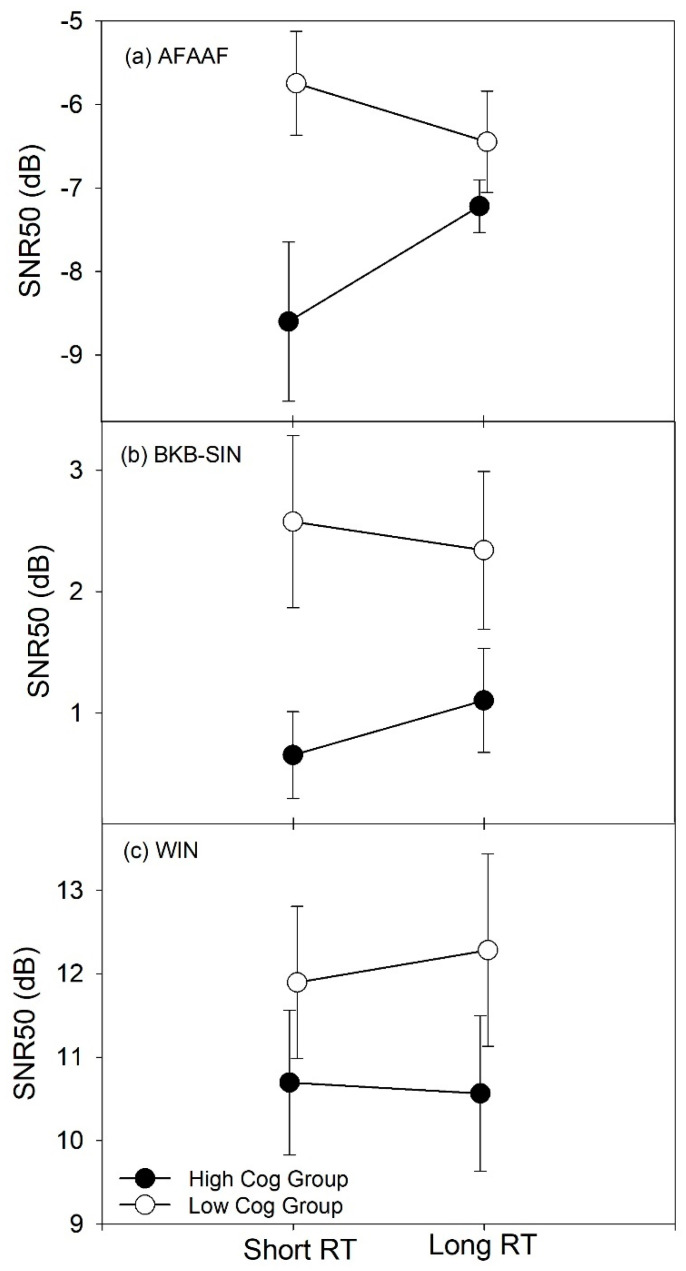
Relationship between cognitive performance groups and RT when using each of the three speech recognition tests. Error bars represent standard errors.

After examining the main effects, the two-way interaction effect between group and RT was examined for each speech recognition test (Figure 8). Inspecting the interaction pattern for each test, the AFAAF test and the BKB-SIN test show a similar pattern. This pattern is also similar to the one with the combined scores shown in Figure 7. However, the WIN test shows a different pattern. With the BKB-SIN and the WIN tests, the SNR50 difference between the two RTs for either group is less than 1 dB. The greatest difference is observed with the AFAAF test, and the difference for either group is less than 2 dB. Taken the slopes of the psychometric functions (Appendix A) into consideration, such SNR50 differences correspond to a difference of a few percent correct in speech recognition performance. Statistical analyses showed that this two-way interaction was only significant for the AFAAF test (F (1, 27) = 4.499, *p* = 0.043), which was due to the difference between the two cognitive groups when using the short RT (*p* = 0.018).

### 3.5. Interaction between RT and Linguistic Context

**Figure 9:**
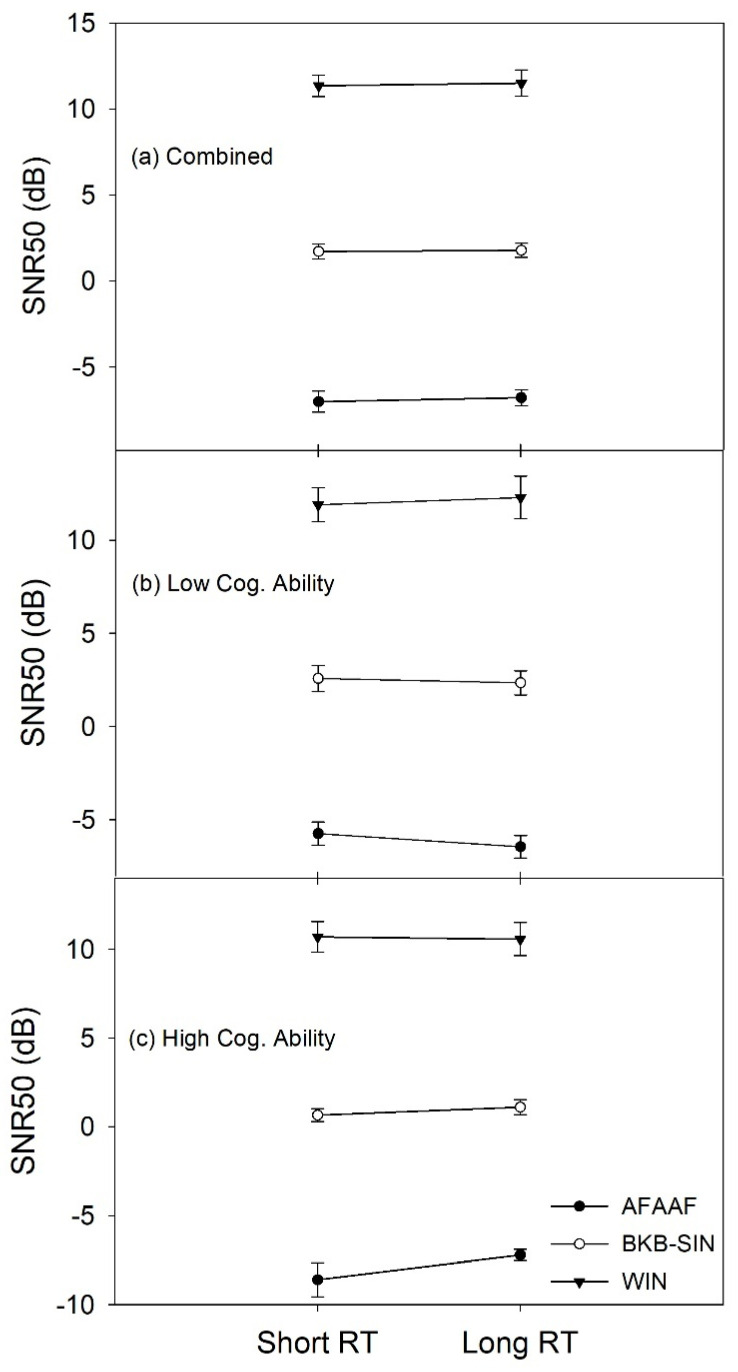
Relationship between RT and speech recognition performance with each test for (**a**) the two cognitive performance groups were combined, (**b**) the low cognitive performance group, and (**c**) the high cognitive performance group. Error bars represent standard errors.

In order to answer the third research question, the effect of linguistic context on RT advantage was examined. Figure 9a shows that the mean SNR50 scores for the two RTs were very similar for each of the three speech recognition tests when the two cognitive groups were combined. The full model ANOVA showed that the two-way interaction effect between RT and context was not significant (Table 2), suggesting that the two RTs did not yield different speech recognition performance with any of the three tests.

In addition, the two-way interaction between RT and context was also examined for each cognitive group. A within-subject design ANOVA was conducted for each of the two cognitive groups. Both RT and context were the within-subject variables. Descriptive data for the two-way interaction analysis are plotted in Figure 9b,c for the two cognitive groups. It can be seen that the mean SNR50 scores for the two RTs were similar for each speech recognition test. The statistical analyses showed that this two-way interaction was not significant for the high cognitive group (F (1.219, 43.448) = 2.719, *p* > 0.1) or the low cognitive group (F (2, 43.448) = 1.042, *p* > 0.1). The results suggested that none of the speech recognition tests produced a significantly different performance between the short and the long RTs for either cognitive group.

## 4. Discussion

The existing research has provided support for the effects of compression RT setting on aided speech recognition performance and has examined the benefits of using short and long RTs. The hearing aids used in previous studies on this topic had two compression channels with the nominal short and long RT settings at 40 ms and 640 ms, respectively. The present study used hearing aids with newer technologies from a different manufacturer using a different processing chip. The hearing aids used in the present study had 12 compression channels with the nominal short and long RT settings at 50 ms and 2000 ms, respectively. The present study is the only known study that used different hearing aid technologies from different manufacturers to assess the relationship between cognitive abilities and RT. The results of the present study, together with the findings reported from the previous studies, make this line of investigation more generalizable across different hearing aids.

### 4.1. Replication of the Result from Gatehouse et al. Study

Gatehouse and colleagues reported a significant correlation of 0.30 between hearing aid users’ cognitive abilities and speech recognition benefit of short over long RT [7]. Based on this finding, they suggested a connection between cognitive abilities and speech recognition performance with different RT processing [7]. They suggested that hearing aid users with higher cognitive abilities had better speech recognition performance with short RT processing. In order to compare with Gatehouse’s finding, the present study adopted the same correlation analyses for each of the three speech recognition tests. Despite the fact that the correlation analyses in the present study were not statistically significant (due to the smaller number of participants), the correlation coefficients were essentially the same magnitude as the one reported in Gatehouse et al.’s [7] study when using the AFAAF test, which is essentially equivalent to the FAAF test used in Gatehouse et al.’s study [7]. Therefore, the present study replicated the result of Gatehouse et al. [7] using an equivalent speech recognition test.

### 4.2. Speech Recognition Performance of Listeners with Different Cognitive Abilities

Previous research has suggested that higher cognitive abilities are associated with better speech understanding performance (e.g., [31]). However, this association was not observed in all existing studies about cognitive ability and speech understanding. This inconsistency has also been found in previous studies examining the relationship between cognitive abilities and aided speech recognition with different RTs. Lunner and Sundewall-Thoren [8] reported a significant main effect of the cognitive group, showing that the cognitively high performing group had significantly better speech recognition performance compared to the cognitively low performing group. However, Gatehouse et al. [6] and Rudner, Foo, Rönnberg, and Lunner [10] did not find the main effect of cognitive group. It is worth noting that the three previous studies used the same cognitive test to quantify hearing aid users’ cognitive abilities but used different speech recognition tests to evaluate their aided speech recognition performance. Using different speech recognition tests in previous research was one factor that was suspected to partially account for these observations.

The present study did not find a significant main effect of cognitive group in the full model ANOVA (Table 3), suggesting that the high cognitive performance group was not significantly different from the low cognitive performance group in aided speech recognition performance, regardless of speech recognition test. However, further analyses of each speech recognition test revealed marginal significant main effects of the cognitive group with the AFAAF and the BKB-SIN tests (Table 3), indicating that hearing aid users with high cognitive abilities have better speech recognition performance compared to their counterparts with low cognitive abilities. This finding supports the suspicion based on the findings from the previous studies that speech recognition tests influence the main effect of cognitive group. The findings from the present study, together with the previous studies, suggest that the effect of cognitive ability on speech recognition performance may depend upon the chosen speech recognition test, at least to some extent.

### 4.3. Relationship between Cognitive Abilities and Speech Recognition with Short and Long RT

The present study examined the interaction effects between cognitive abilities, RT, and linguistic context to explore the relationship between cognitive abilities and aided speech recognition performance in noise with different RTs. The results of the interaction between cognitive ability and RT indicated that hearing aid users with high cognitive abilities performed better with a short RT than with a long RT, irrespective of speech recognition test (Table 2 and Figure 7). However, the present study found no significant effect of RT on speech recognition performance for either cognitive group with any of the three tests (Figure 8). In addition, the results of the interaction between linguistic context and RT showed that hearing aid users as a group did not have significantly different speech recognition performance between the short and the long RTs when using any of the three tests (Figure 9a). Moreover, none of the speech recognition tests produced significantly different performance between the two RTs for either cognitive group (Figure 9b,c).

In the present study, hearing aid users with high cognitive abilities performed significantly better than did their counterparts with low cognitive abilities when using short RT processing (Figure 8). This pattern was only observed for the AFAAF test. This suggested that the association between high cognitive abilities and short RT processing may only hold when using certain types of speech recognition tests. To examine this factor, the present study incorporated three speech recognition tests with different amounts of linguistic context. The BKB-SIN test and the WIN test are both open-set tests categorized as the context-high and the context-low tests, respectively. As described earlier, the BKB-SIN test is a sentence test and allows for substantial top-down processing to understand words and sentences. However, the WIN test is a word-based test. Listeners must rely on bottom-up processing to understand the test words because the linguistic context in the WIN test is limited. The AFAAF test is very different from the BKB-SIN test and the WIN test. First, it is a closed-set word-based test with a fixed carrier phrase. Second, the displayed alternatives differ in one or two phonological features (minimal pair), which can be very confusing. Considering the characteristics of the AFAAF test, it is assumed that some predictive cues from the displayed alternatives are available to listeners to assist in speech recognition. Therefore, in the planning phase of the present study, the AFAAF test was considered an intermediary between the BKB-SIN test and the WIN test. Assuming an effect of linguistic context on the relationship between cognitive abilities and RT, speech recognition performance with the three tests should reveal a pattern that would follow the order of the amount of linguistic context or predictive cues. However, the findings from the present study did not show such a pattern. Instead, the AFAAF test and the BKB-SIN test showed the same pattern regarding the relationship between cognitive abilities and speech understanding with short and long RTs, while the WIN test showed a pattern that was opposite to the other two speech recognition tests. It is also interesting that the AFAAF test produced the greatest effect on the difference between the two cognitive groups. Moreover, the BKB-SIN test did not differ substantially from the WIN test in patterns of speech recognition performance in terms of the factors of cognitive ability and RT (Figure 9b,c).

The AFAAF test and the BKB-SIN test produced similar speech recognition patterns, suggesting that these two tests affected the relationship between cognitive abilities and speech understanding with different RTs the same way. This is probably because both speech recognition tests allow top-down processing to facilitate speech understanding in addition to audibility. For the BKB-SIN test, the top-down processing is involved probably because of its context-rich test sentences. Regarding speech recognition, working memory is a capacity-limited system that both stores recent phonological information in short-term memory and simultaneously processes the information with knowledge stored in long-term memory. With the BKB-SIN test, working memory capacity is linked to speech understanding. However, since the contextual cues are involved in speech understanding, RT may become less important [4]. The AFAAF test engages top-down processing via a certain amount of predictive cues from displayed alternatives. Notably, the AFAAF test produced the greatest effect on the difference between the two cognitive groups. This suggests that the AFAAF test has some other unique characteristics in addition to linguistic context, which makes it sensitive to differences in cognitive abilities. This may also explain why Gatehouse’s study, which used the FAAF test, could not be replicated by some similar studies. Later sections offer further discussion about the characteristics of the AFAAF test and their possible impacts on speech understanding.

In contrast to the AFAAF test and the BKB-SIN test, the WIN test is an open-set monosyllabic word test that has limited linguistic context. Understanding the test words masked by noise mostly relies on audibility. Thus, speech recognition is mainly based on bottom-up processing. Consequently, cognitive abilities may not be involved as much as with the other two speech recognition tests. It is interesting that the short and the long RT processing did not reveal substantial and statistically significant differences in speech recognition performance for either group for both the BKB-SIN test and the WIN test (Figure 9b,c). This implies that a hearing aid user’s speech recognition performance in noise with different RT settings probably does not depend on the linguistic context of speech recognition test material. Thus, it can be argued that the linguistic context of speech recognition tests may not be considered a critical factor in assessing the relationship between cognitive abilities and understanding speech with different RTs. The results from the present study do not support the hypothesis proposed by Cox and Xu [4]. Surprisingly, the AFAAF test produced the greatest difference between the two cognitive groups. It is reasonable to speculate that other factors embedded in speech recognition tests influence the assessment of the relationship of interest.

### 4.4. What Makes the AFAAF a Sensitive Test to Detect Cognitive Difference?

The AFAAF test is a word-based closed-set test. Fundamental and underlying differences exist between open-set and closed-set tests, in addition to chance performance and the way the two types of tests are administered. Clopper, Pisoni, and Tierney [32] suggested that crucial differences between the two test formats are due to the nature of the specific task demands imposed on lexical access of phonetically similar words. In open-set tests, listeners must compare the target word to all possible candidate words in their lexical memories, whereas in closed-set tests, the listeners only need to make a limited number of comparisons using the provided options. Therefore, the difference in task demand of the two test formats is, in essence, due to differences in lexical competition effect.

At first glance, it seems obvious that recognizing the same words requires more word comparisons in open-set tests compared to closed-set tests. As a result, open-set tests are more difficult compared to closed-set tests. However, lexical competition in closed-set tests could increase if the degree of confusion between response alternatives increased [32]. According to Clopper et al. [32], greater phonetic confusability among the response alternatives is associated with greater lexical competition in a closed-set test because it requires an effort from a listener to differentiate subtle phonetic differences.

Phonemes are confusable when they are phonologically similar. When the task is to distinguish the target item from several phonologically similar items, the probability of losing a phonological feature, which discriminates the target item from other items of the memory set, will be greatest when the number of discriminating features is small [33]. For example, it is difficult to distinguish the target word from the given minimal pair of response alternatives because only one or two phonemes differentiate a pair of words. Comparing phonologically similar words can affect short-term memory (STM). Evidence shows that the similarity between phonemes leads to STM confusions of English vowels and consonants [34,35].

In addition to confusable phonemes, visual information about the displayed response alternatives is stored in a listener’s short-term memory for comparison. It has been proposed that this visual information relates to a listener’s cognitive ability, although there is little direct evidence to support this proposition. Previous research on visual word recognition shed some light on this subject. Gathercole and Baddeley [36] reviewed studies on visual word recognition performance and suggested an association with working memory when certain types of word recognition tasks (e.g., rhyme judgment) are required. Comparing two phonological representations requires information storage, which can impose a substantial memory load.

As described earlier, working memory includes short-term memory (temporal storage) and other processing mechanisms (central executive) that help make use of short-term memory [13,37]. The confusion induced by similar phonemes in short-term memory could potentially influence the function of working memory. In addition, reading phonologically similar response alternatives may increase a hearing aid user’s working memory load. Therefore, since working memory is one aspect of cognitive processing [13], it is likely to be heavily involved in distinguishing the target word from the non-target alternatives when they are phonologically similar to each other. Therefore, it is reasonable to argue that listeners with higher cognitive abilities could differentiate similar phonemes and identify the target words more accurately compared to their counterparts with lower cognitive abilities.

According to the above discussion, among the three speech recognition tests in the present study, the AFAAF test appears to be the most sensitive test in detecting differences in cognitive ability. One of the reasons is that the four alternatives on the test are constructed based on a minimal pair structure, while the alternatives are very similar in terms of phonemes. Thus, the lexical competition aimed at differentiating subtle phonological differences between target words and the corresponding non-target alternatives is high. This requires a higher cognitive capability to process the confusable lexical information stored in short-term memory. Moreover, the minimal pair structure for the alternatives also makes storage and comparison of the displayed alternatives demanding, requiring higher cognitive ability. Consequently, when using the AFAAF test, speech recognition performance could be considerably better among listeners with higher cognitive abilities.

Because the BKB-SIN test and the WIN test are two open-set tests, listeners are required to compare the target words to all possible candidate words in their lexical memories. These two tests differ substantially in their amount of linguistic context. For the BKB-SIN test, it is probable that the effect of linguistic context overcomes the effect of lexical competition, as the linguistic context is substantial. In contrast, the WIN test has limited linguistic context, and the lexical competition is truly determined based on the comparisons with the listener’s lexical memory. Therefore, in the present study, it is not appropriate to classify the AFAAF test as an intermediary test among the three speech recognition tests in terms of linguistic context. It is clear that the AFAAF test is very different from the other two tests.

### 4.5. What RT Should Be Prescribed?

One of the clinically relevant questions that remain unclear is what compression RT, a shorter one or a longer one, should be prescribed for hearing aid users. Previous research has suggested that a hearing aid user’s cognitive ability is one possible predictor, which can assist clinicians in selecting an appropriate compression RT. However, the results were not consistent. The results from the present study failed to indicate statistically significant benefits with short or long RT for either cognitive group. It is seen from Table 1 that the two mean SNR50 values for each speech recognition test are similar, suggesting that adjusting release time is neither beneficial nor detrimental when listening to speech in noise. Therefore, at this moment, either a short or a long RT could be prescribed for hearing aid users, regardless of their cognitive abilities.

Nonetheless, this recommendation is still open to debate. The results from the present study suggest that the selection of the most advantageous RT for a given hearing aid user might be dependent on factors other than cognitive ability.

### 4.6. Limitations

There are some limitations to this study. First, in addition to linguistic context, listeners may use other cues, such as coarticulation cues, prosodic cues, and prior knowledge about the topic, to assist in understanding speech when listening to sentences or discourses (e.g., [38]). The effect of these cues was not controlled in the present study. Second, aided speech recognition performance measured in the present study may be impacted by WDRC. When using different speech recognition test materials and SNRs, WDRC amplifiers could behave differently due to the varying amplitude of the test stimuli, resulting in various gains for keywords in a sentence test versus in a word-based test. The impact of WDRC may be especially pronounced for the AFAAF and the WIN tests because both of them use a constant carrier phrase and place the keyword at the same place. Therefore, the effects of the WDRC might not have been the same for the three speech recognition tests used in this study. Although it is beyond the scope of the present study, some acoustic measurements, for example, the temporal envelope of the hearing aid outputs for the three tests, can, to some extent, facilitate the evaluation of the impact of WDRC. Changes in temporal envelopes of amplified speech in the presence of noise will result in different output SNRs when using different compression time constants [39], which will have an impact on audibility and how much speech cues can be utilized for top-down and bottom-up processing. Future research regarding the effect of linguistic context of speech recognition tests should take WDRC behavior into consideration.

## 5. Conclusions

The present study contributes to the growing body of research on the assessment of the relationship between hearing aid users’ cognitive abilities and their speech recognition performance with short and long RTs. Results from Gatehouse’s study were reproduced in this study when using an equivalent speech recognition test. Results obtained from this study showed that none of the speech recognition tests produced significantly different performance between the short and the long RTs for either cognitive group. Based on results with high-context and low-context tests, the linguistic context of speech recognition tests may not be a critical factor in speech understanding with different RTs. Other factors of aided speech recognition testing may associate with the assessment of the relationship of interest. Regarding a recommendation of prescribed RT, it is argued that either a short or a long RT could be prescribed for hearing aid users regardless of their cognitive abilities because the obtained SNR differences in the present study between the two RT settings were small and probably not clinically important. This recommendation is open to debate. Results from the present study also suggest that cognitive ability is associated with speech understanding in noise for hearing aid users, but it may not be important in prescribing RT.

## Figures and Tables

**Table 1:** Mean SNR50 scores and SDs for the three speech recognition tests (N = 34).

Speech Recognition Test	Release Time	SNR50 (dB)	
		Mean	SD
AFAAF	Short	−6.92	3.15
	Long	−6.81	2.37
BKBSIN	Short	1.60	2.22
	Long	1.62	2.01
WIN	Short	11.19	3.33
	Long	11.33	3.98

**Table 2:** Effects of release time (RT), cognitive abilities (group), and linguistic context (context) on aided speech recognition performance in the full model ANOVA.

Effects	dfs	F	*p*
Group	1, 27	3.119	0.089
RT	1, 27	1.055	0.313
Context	2, 54	826.004	<0.001
Group * RT	1, 27	4.023	0.055
Group * Context	2, 54	0.079	0.924
RT * Context ^a^	1.609, 43.448	0.131	0.834
Group * RT * Context ^a^	1.609, 43.448	3.303	0.056

^a^ with Greenhouse–Geisser adjustment.

**Table 3:** Mean SNR50 (dB) for the two RTs and the two cognitive performance groups. Values enclosed in parentheses represent one standard deviation (N = 29).

	Release Time		Cognitive Group	
	Short	Long	Low	High
AFAAF	−7.17 (3.04)	−6.83 (2.56)	−6.10 (2.47)	−7.91 (2.47)
BKB-SIN	1.62 (2.21)	1.72 (2.14)	2.46 (2.07)	0.88 (2.07)
WIN	11.30 (3.40)	11.42 (4.09)	12.09 (3.68)	10.63 (3.68)

**Figure A1:**
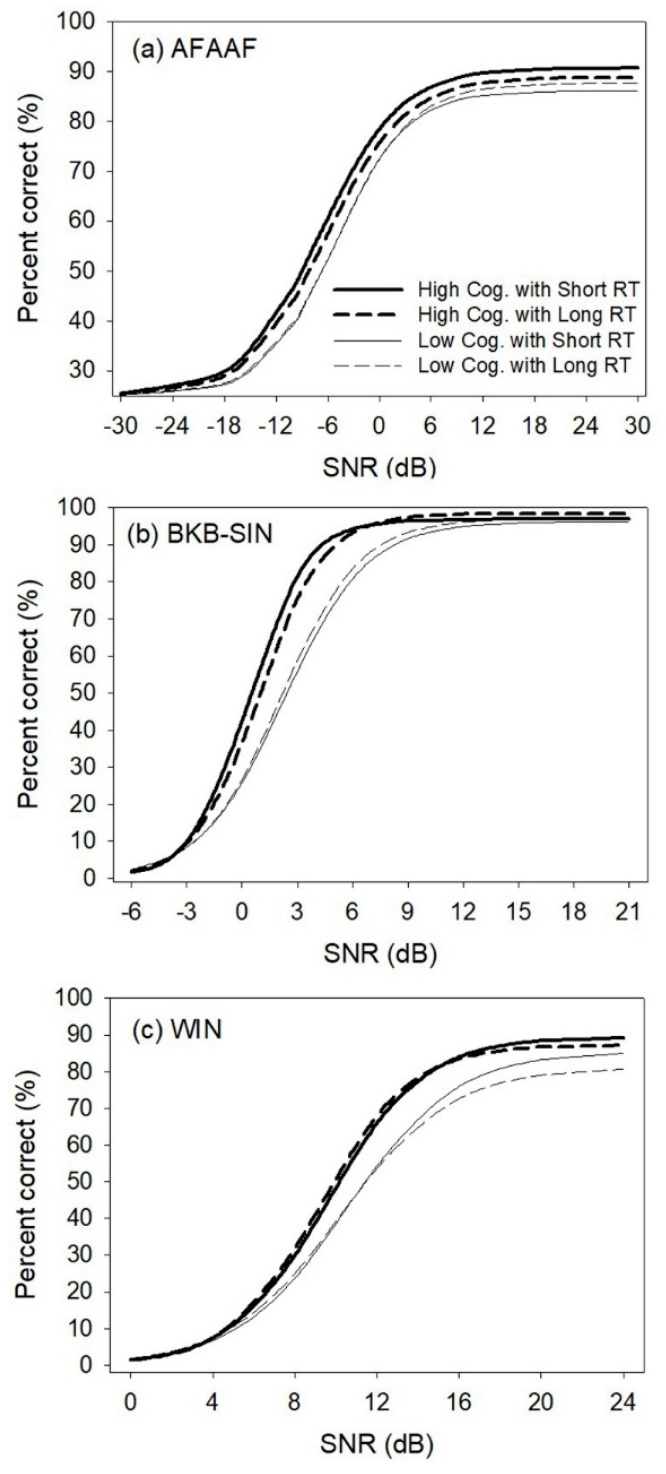
Psychometric functions were fitted to the mean percent correct scores for each cognitive group and each speech recognition test with both RTs using Equations (1) and (2) for displaying performance patterns. The slopes for high and low cognitive groups are 13%/dB and 8%/dB for the BKB-SIN test and 10%/dB and 7%/dB for the WIN test, respectively. With the AFAAF test, slopes for both cognitive groups are about 3%/dB. Tested SNRs were from −9 to +12 dB for the AFAAF test.

## Data Availability

The data presented in this study are available on request from the corresponding author.

## References

[1] Moore B.C.J. (2008). The choice of compression speed in hearing aids: Theoretical and practical considerations and the role of individual differences. Trends Amplif..

[2] Bentler R.A., Nelson J.A. (1997). Assessing release-time options in a two-channel AGC hearing aid. Am. J. Audiol..

[3] Gilbert G., Akeroyd M.A., Gatehouse S. (2008). Discrimination of release time constants in hearing-aid compressors. Int. J. Audiol..

[4] Cox R.M., Xu J. (2010). Short and long compression release times: Speech understanding, real-world preferences, and association with cognitive ability. J. Am. Acad. Audiol..

[5] Foo C., Rudner M., Ronnberg J., Lunner T. (2007). Recognition of speech in noise with new hearing instrument compression release settings requires explicit cognitive storage and processing capacity. J. Am. Acad. Audiol..

[6] Gatehouse S., Naylor G., Elberling C. (2003). Benefits from hearing aids in relation to the interaction between the user and the environment. Int. J. Audiol..

[7] Gatehouse S., Naylor G., Elberling C. (2006). Linear and nonlinear hearing aid fittings—2. Patterns of candidature. Int. J. Audiol..

[8] Lunner T., Sundewall-Thoren E. (2007). Interactions between cognition, compression, and listening conditions: Effects on speech-in-noise performance in a two-channel hearing aid. J. Am. Acad. Audiol..

[9] Reinhart P.N., Souza P.E. (2016). Intelligibility and clarity of reverberant speech: Effects of wide dynamic range compression release time and working memory. J. Speech Lang. Hear. Res..

[10] Rudner M., Foo C., Rönnberg J., Lunner T. (2009). Cognition and aided speech recognition in noise: Specific role for cognitive factors following nine-week experience with adjusted compression settings in hearing aids. Scand. J. Psychol..

[11] Rudner M., Ronnberg J., Lunner T., Rönnberg J. (2011). Working memory supports listening in noise for persons with hearing impairment. J. Am. Acad. Audiol..

[12] Akeroyd M.A. (2008). Are individual differences in speech reception related to individual differences in cognitive ability? A survey of twenty experimental studies with normal and hearing-impaired adults. Int. J. Audiol..

[13] Baddeley A.D., Hitch G.J. (1974). Working memory. Psychol. Learn. Motiv..

[14] Pichora-Fuller M.K., Schneider B.A., Daneman M. (1995). How young and old adults listen to and remember speech in noise. J. Acoust. Soc. Am..

[15] Foster J.R., Haggard M.P. (1987). The four alternative auditory feature test (FAAF)-linguistic and psychometric properties of the material with normative data in noise. Br. J. Audiol..

[16] Massaro D.W. (1994). Psychological aspects of speech perception: Implications for research and theory. Handbook of Psycholinguistics.

[17] Pichora-Fuller M.K., Singh G. (2006). Effects of age on auditory and cognitive processing: Implications for hearing aid fitting and audiologic rehabilitation. Trends Amplif..

[18] Byrne D., Dillon H., Ching T., Katsch R., Keidser G. (2001). NAL-NL1 procedure for fitting nonlinear hearing aids: Characteristics and comparisons with other procedures. J. Am. Acad. Audiol..

[19] Daneman M., Carpenter P.A. (1980). Individual differences in working memory and reading. J. Verbal Learn. Verbal Behav..

[20] Rönnberg J., Arlinger S., Lyxell B., Kinnefors C. (1989). Visual evoked potentials: Relation to adult speechreading and cognitive function. J. Speech Lang. Hear. Res..

[21] Wilson R.H. (2003). Development of a speech-in-multitalker-babble paradigm to assess word-recognition performance. J. Am. Acad. Audiol..

[22] Xu J., Cox R. (2014). Recording and Evaluation of an American Dialect Version of the Four Alternative Auditory Feature Test. J. Am. Acad. Audiol..

[23] Tillman T.W., Carhart R. (1966). An Expanded Test for Speech Discrimination Utilizing CNC Monosyllabic Words: Northwestern University Auditory Test No. 6.

[24] Wilson R.H., McArdle R.A., Smith S.L. (2007). An evaluation of the BKB-SIN, HINT, QuickSIN, and WIN materials on listeners with normal hearing and listeners with hearing loss. J. Speech Lang. Hear. Res..

[25] Dreschler W.A., Verschuure H., Ludvigsen C., Westermann S. (2001). ICRA Noises: Artificial Noise Signals with Speech-like Spectral and Temporal Properties for Hearing Instrument Assessment. Int. J. Audiol..

[26] Rönnberg J. (2003). Cognition in the hearing impaired and deaf as a bridge between signal and dialogue: A framework and a model. Int. J. Audiol..

[27] Füllgrabe C., Rosen S. (2016). On the (un) importance of working memory in speech-in-noise processing for listeners with normal hearing thresholds. Front. Psychol..

[28] Cohen J. (1988). Statistical Power Analysis for the Behavioral Sciences.

[29] Faul F., Erdfelder E., Lang A.-G., Buchner A. (2007). G*Power 3: A flexible statistical power analysis program for the social, behavioral, and biomedical sciences. Behav. Res. Methods.

[30] Tabachnick B.G., Fidell L.S. (2006). Using Multivariate Statistics: International Edition.

[31] Lunner T. (2003). Cognitive function in relation to hearing aid use. Int. J. Audiol..

[32] Clopper C.G., Pisoni D.B., Tierney A.T. (2006). Effects of open-set and closed-set task demands on spoken word recognition. J. Am. Acad. Audiol..

[33] Salamé P., Baddeley A. (1982). Disruption of short-term memory by unattended speech: Implications for the structure of working memory. J. Verbal Learn. Verbal Behav..

[34] Wickelgren W.A. (1965). Distinctive Features and Errors in Short-Term Memory for English Vowels. J. Acoust. Soc. Am..

[35] Wickelgren W.A. (1966). Distinctive Features and Errors in Short-Term Memory for English Consonants. J. Acoust. Soc. Am..

[36] Gathercole S.E., Baddeley A.D. (1993). Working Memory and Language.

[37] Cowan N. (2008). What are the differences between long-term, short-term, and working memory?. Prog. Brain Res..

[38] Greenberg S., Arai T. (2004). What are the essential cues for understanding spoken language?. IEICE Trans. Inf. Syst..

[39] Naylor G., Johannesson R.B. (2009). Long-term signal-to-noise ratio at the input and output of amplitude-compression systems. J. Am. Acad. Audiol..

